# Possible COVID-19-Associated Pulmonary Aspergillosis Due to *Aspergillus niger* in Greece

**DOI:** 10.3390/antibiotics11030300

**Published:** 2022-02-23

**Authors:** Maria Katsiari, Angeliki Mavroidi, Eleftheria Palla, Konstantina Zourla, Theodoros Alonistiotis, Kyriakos Ntorlis, Charikleia Nikolaou, Georgia Vrioni, Athanasios Tsakris

**Affiliations:** 1 Intensive Care Medicine, General Hospital of N. Ionia Konstantopouleio-Patission, 14233 Athens, Greece; mkatsiari71@gmail.com (M.K.); alonisteo@gmail.com (T.A.); ntorlikos@yahoo.gr (K.N.); hariklia2009@yahoo.gr (C.N.); 2 Department of Microbiology, General Hospital of N. Ionia Konstantopouleio-Patission, 14233 Athens, Greece; amavroidi@live.com (A.M.); elefthepal@gmail.com (E.P.); kzourla@yahoo.gr (K.Z.); 3 Department of Microbiology, Medical School, National and Kapodistrian University of Athens, 11527 Athens, Greece; gvrioni@med.uoa.gr

**Keywords:** COVID-19-associated pulmonary aspergillosis, ICU, *Aspergillus niger*, voriconazole, amphotericin B

## Abstract

Severe acute respiratory syndrome coronavirus 2 (SARS-CoV-2) causes direct damage to the pulmonary epithelium, enabling *Aspergillus* invasion. Rapid progression and high mortality of invasive aspergillosis have been reported. In the present study, we report a rare case of possible COVID-19-associated pulmonary aspergillosis (CAPA) caused by *A. niger* in a Greek patient. Diagnosis was based on ECMM/ISHAM specific criteria and the new algorithm “BM-AspICU” for the invasive pulmonary aspergillosis diagnostic strategy. The fungal isolate was recovered in a non-bronchoalveolar lavage (non-BAL) sample and its identification was performed by standard macroscopic and microscopic morphological studies. MALDI-TOF analysis confirmed the identification of *A. niger*. In addition, galactomannan antigen and *Aspergillus* real-time PCR testing were positive in the non-BAL sample, while in serum they proved negative. The *A. niger* isolate showed an MIC for fluconazole ≥128 μg/mL, for itraconazole and posaconazole 0.25 μg/mL, for voriconazole 0.5 μg/mL, for flucytosine 4 μg/mL, for amphotericin B 1 μg/mL, and for all echinocandins (caspofungin, anidulafungin, micafungin) >8 μg/mL. The patient was initially treated with voriconazole; amphotericin B was subsequently added, when a significant progression of cavitation was demonstrated on chest computed tomography. *A. niger* was not isolated in subsequent samples and the patient’s unfavorable outcome was attributed to septic shock caused by a pandrug-resistant *Acinetobacter baumannii* strain.

## 1. Introduction

*Aspergillus* conidia are ubiquitous in the environment, and upon exposure and inhalation may invade the human pulmonary system [1]. Severe acute respiratory syndrome coronavirus-2 (SARS-CoV-2) causes direct damage to the pulmonary epithelium, enabling *Aspergillus* invasion [2]. Therefore, COVID-19 pneumonia per se has been suggested as a possible risk-factor for COVID-19-associated pulmonary aspergillosis (CAPA). Moreover, immune dysregulation associated with COVID-19 involves hyperactivation of IL-1 and IL-6 and decrease in T-cell populations [2,3], and treatment with anti-IL-6 agents, such as tocilizumab and corticosteroids, can also predispose to CAPA [4,5]. In addition, chronic cardiovascular disease, renal failure, diabetes mellitus, and corticosteroid use have been identified as risk-factors among patients with CAPA [6]. 

Obtaining a diagnosis can be challenging, since patients with CAPA might not have host factors and typical clinical and radiological features, whereas mycological evidence is mostly based on positive *Aspergillus* cultures in non-bronchoalveolar lavage (non-BAL) respiratory samples, which may reflect colonization rather than infection [7,8]. CAPA-specific diagnostic criteria have been recently developed by the European Confederation of Medical Mycology (ECMM) and the International Society for Human and Animal Mycology (ISHAM) [9]. ECMM/ISHAM consensus criteria permit diagnosis of possible pulmonary aspergillosis based on microscopic detection or culture of fungus in non-BAL respiratory samples, as mycological evidence criterion [9]. Recently, a new algorithm “BM-AspICU” (biomarkers-*Aspergillus* in Intensive Care Unit) has been proposed to be systematically part of the invasive pulmonary aspergillosis (IPA) diagnostic strategy [10]. The BM-AspICU algorithm includes fungal biomarkers, such as galactomannan (GM) antigen detection and *Aspergillus* qPCR, in intensive care unit (ICU) patients without risk-factors (e.g., immunosuppression from organ transplant or neutropenia) as mentioned in revised criteria for invasive fungal infections from the European Organization for the Research and Treatment of Cancer/Mycosis Study Group Education and Research Consortium (EORTC/MSGERC) [11]. Nonetheless, the inclusion of biomarkers, such as *Aspergillus* molecular detection, to identify probable IPA needs validation in the ICU population [12].

Concerning antifungal treatment, either voriconazole or isavuconazole are recommended as first-line therapy for CAPA, while liposomal amphotericin B is the primary alternative option for ICU patients [3]. Previous studies have reported a non-statistically significant lower mortality rate among CAPA patients who were treated with voriconazole [6,13]. Reported CAPA mortality is exceeding 50%, although attributed mortality can be substantially lower (17–27%), especially in the setting of bacterial infections [6,13,14,15]. 

The exact incidence of CAPA in COVID-19 patients is not known and presents great variation, ranging from 3.3% to 33.3% [13,14,16,17,18,19,20]. Identification findings from BAL samples have shown that CAPA is mainly associated with *A. fumigatus* and *A. flavus*, while *A. niger* has been occasionally recovered from limited geographical regions [13,14,15,16]. In this study, we report a possible CAPA due to *A. niger* in a Greek patient. 

## 2. Methods and Case Study

A 70-year-old man, with clinical history of diabetes and arterial hypertension, was admitted in our hospital with a 6-day fever and dyspnea due to COVID-19. He was intubated within the first 24 h and transferred to the ICU. Upon admission, he presented severe respiratory insufficiency (PaO_2_/FiO_2_ < 100 mm Hg), hemodynamic stability, normal white blood cell count (7500/mm^3^), and markedly elevated levels of inflammatory markers (C-reactive protein = 333 mg/dL; procalcitonin = 42 μg/L; ferritin = 1500 ng/mL). Cultures of tracheal aspirates and blood were negative, whereas pneumococcal urine antigen test was positive. His initial treatment included corticosteroid therapy with dexamethasone 6 mg/day, remdesivir and ceftriaxone. The timeline of the methods and results for diagnosis, and therapeutic regimens applied in the case study are summarized in Table 1.

**Figure 1:**
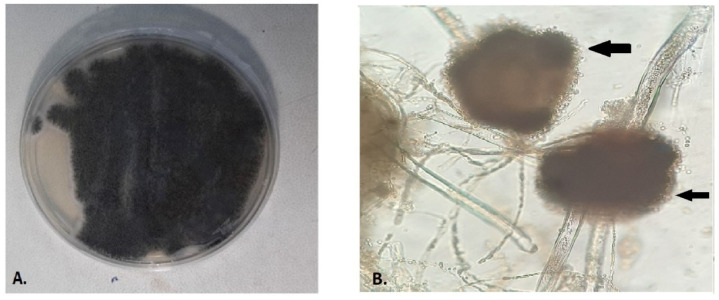
Growth of *Aspergillus* spp. on Sabouraud Dextrose agar at 37 °C after 48 h (**A**) and microscopic examination of the cultured fungus at 40× magnification (some fungal colonies were picked up with adhesive tape, placed on clean glass slide, and covered with a slip). The arrows indicate the conidiophore with phialides covering its entire surface (**B**).

On day 5, *Aspergillus* spp. and *Candida albicans* were isolated from tracheal aspirates (Figure 1). On day 6, non-BAL was performed and GM antigen (GM immunoenzymatic Platelia *Aspergillus*, Bio-Rad, Hercules, CA, USA) and real-time PCR assay (*Aspergillus* PCR) for the detection of *Aspergillus* spp. (Standard Real-Time PCR detection kit for *Aspergillus*, Primerdesign™Ltd, genesig^®^ kit) were carried out. Simultaneously, GM antigen and *Aspergillus* PCR were performed in patient’s serum sample. GM antigen and *Aspergillus* PCR were positive in non-BAL sample, while in serum they proved negative, as previously reported in COVID-19 patients [21]. Identification of the *A. niger* isolate was performed by standard phenotypic methods, based on macroscopic and microscopic morphological studies [22]. Matrix-assisted laser desorption ionization time of flight mass spectrometry (MALDI-TOF) on a Microflex LT (Bruker Daltonics, Bremen, Germany) platform confirmed the identification of the isolate as *A. niger*.

**Figure 2:**
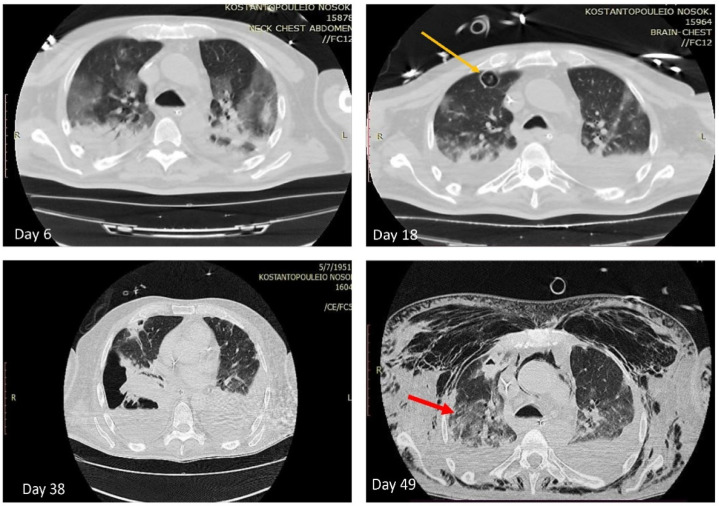
Chest computed tomography (CT) showing multi-lobar peripheral ground-glass opacities and consolidations (day 6); the yellow arrow depicts one cavitary lesion (day 18); the red arrow depicts the bronchopleural fistula (day 49).

Susceptibility to antifungal agents was evaluated by the EUCAST standardized broth microdilution method (*Aspergillus* spp. EUCAST Antifungal Clinical Breakpoints Table v. 10.0 valid from 4 February 2020) [23]. MIC values of ≥128 μg/mL for fluconazole, 0.25 μg/mL for itraconazole, 0.5 μg/mL for voriconazole, 0.25 μg/mL for posaconazole, 4 μg/mL for flucytosine, 1 μg/mL for amphotericin B, and >8 μg/mL for all echinocandins (caspofungin, anidulafungin, micafungin) were obtained. The patient was administrated with voriconazole on day 6 and computed tomography (CT) was performed (Figure 2), which revealed bilateral ground glass opacities and consolidations of dependent parts of lung parenchyma.

On day 11, surveillance sample of tracheal aspirates was tested with FilmArray^®^, PneumoniaPanel*plus* (BIOFIRE, Biomerieux, France) and revealed the following pathogens (copies/mL): *Acinetobacter calcoaceticus-baumannii* complex (≥10^7^), *Streptococcus pneumoniae* (10^5^), KPC-producing *Klebsiella pneumoniae* group (10^4^), *Staphylococcus aureus* (10^4^). Culture of the same sample identified only *A. baumanni* by the Vitek^®^ Compact15 (Biomerieux) and the antimicrobial susceptibility testing revealed a pan-drug resistant strain. Therefore, ceftriaxone was replaced by meropenem plus colistin. 

Being on treatment, another CT scan was performed on day 18 (Figure 2), which disclosed three cavitary lesions with diameter up to 2.2 cm at upper and middle lobe of the right lung and liposomic amphotericin B was added for treatment. At serial CT scans the cavitary lesions remained unchanged, while the patient presented pulmonary embolism (day 26), pyopneumothorax (day 38), and bronchopleural fistula (day 49) (Figure 2). Culture of the corresponding pleural effusion (day 38) revealed only the pandrug-resistant *A.* *baumannii* and the patient started on another course of meropenem, tigecycline, and colistin. Notably, *A. niger* was not isolated from subsequent samples of tracheal aspirates. However, his weaning from mechanical ventilation was not accomplished, due to damage of lung parenchyma along with critical illness polyneuropathy and myopathy and tracheostomy was performed. Moreover, his clinical condition deteriorated and finally he died on day 60, due to septic shock and multi-organ failure. 

Due to the CAPA case a surveillance study was undertaken in the ICU. Bronchial secretions were obtained twice per week from ICU patients. *A. fumigatus* was identified by MALDI-TOF in cultures from tracheal aspirates of two other contemporary patients, but represented colonization. Moreover, cultures of environmental samples obtained from the bedsides of the ICU patients revealed *A. fumigatus*, *A. versicolor* and *A. mondevidensis* by MALDI-TOF.

## 3. Discussion

Early diagnosis and treatment can affect the rapid progression of invasive aspergillosis and substantially patients’ prognosis and survival. Although bronchoscopy allows direct inspection of trachea and bronchi and enables the clinician to obtain BAL and lung biopsy samples, this practice is avoided in COVID-19 patients due to its nature of aerosol generation and high-risk of viral transmission to health-care workers [7]. Respiratory samples are the preferred specimens for fungal diagnostics. Nevertheless, in COVID-19 patients, diagnosis is often delayed due to lack of clinical recognition and typical radiological features [16,17,20,21]. Differentiation between colonization and invasive aspergillosis is often challenging since most COVID-19 patients are immunocompetent [14,21]. 

In the present study, the early diagnosis of a possible CAPA case was accomplished by the combination of morphological characteristics of cultures with molecular methods, such as PCR detection of *Aspergillus* spp., identification to the species level (*A. niger*) by MALDI-TOF and detection of the antigen GM in non-BAL respiratory samples. CAPA diagnosis was established after 6 days of ICU stay and after 12 days after COVID-19 symptoms onset, which are in accordance with previous studies [15,16,18,19,20,24]. Therefore, the case was classified as possible invasive pulmonary aspergillosis by clinical, radiological, and mycological criteria according to ECMM/ISHAM and the BM-AspICU criteria [9,10].

The patient has been hospitalized in the nine-bed ICU of the hospital after the third wave of COVID-19 that occurred in Greece and was the only patient diagnosed with CAPA among 90 COVID-19 patients at that time point, resulting in an incidence of 1.1%. The incidence of CAPA in our ICU is considerably lower in comparison to other studies [20,21,25,26], but similar with the first report CAPA ICU cases in Greece [15]. Incidence differences are observed between countries attributed to geographical differences along with different risk-factors and applied diagnostic criteria [16].

*A. fumigatus* is a much more common cause of invasive pulmonary infection compared to *A. niger*, probably due to their differences in pathogenicity. Indeed, in a retrospective analysis of 186 CAPA patients [6] and a review of 86 cases [13], the most common pathogen was *A. fumigatus*, whereas in the first report regarding Greek CAPA ICU cases, the isolated pathogens included *A. fumigatus, A. flavus,* and *A. terreus* [15]. Reported CAPA mortality in the literature is exceeding 50%, although attributed mortality can be substantially lower (17–27%), especially in the ICU setting of bacterial infections [13,14,15,27]. On the contrary, in a case series of eight patients with invasive *A. niger* infection and hematological malignancies, three were on high-dose steroids and the attributed mortality accounted for 75% [28].

Antifungal-resistant pathogens appear to be increasing in frequency, especially in medical centers attending patients with complex underlying disease, such as immunocompromised patients [29,30,31,32]. Inadequate fungal treatment (delayed or not given) may lead to poor outcomes, thus routine antifungal susceptibility testing and its adequate interpretation is necessary for the prudent use of antifungal agents. Accordingly, based on the identification of *A. niger* and the susceptibility to antifungal agents, the reported case received initially voriconazole. However, he demonstrated significant progression of cavitation on chest CT despite therapy. One possible explanation could be azole resistance, but based on the strain’s MIC for voriconazole, this was the outmost scenario. Another possible explanation for progressive disease could be that subtherapeutic voriconazole levels were contributing to treatment failure. Considering that monitoring of voriconazole serum levels was not feasible, amphotericin B was added for therapy. Nevertheless, *A. niger* was not isolated again in subsequent samples and his unfavorable outcome was attributed to septic shock caused by the pandrug-resistant *A. baumannii.*

In conclusion, we reported a rare possible case of CAPA due to *A. niger* in a Greek ICU patient with severe pneumonia, who was immunocompromised due to COVID-19 treatment. Although the definite diagnosis lacks histopathological confirmation, the reported case demonstrated the application of 2020 ECMM/ISHAM and the BM-AspICU criteria in this case, along with the need for early identification and therapeutic drug monitoring of voriconazole. Clinicians need to have a high level of vigilance since delayed CAPA diagnosis might compromise patients’ prognosis and outcome.

## Figures and Tables

**Table 1:** Timeline of the methods, results and therapeutic regimens applied in the case study.

Length of Stay (Days) in the ICU	Methods	Results	Therapeutic Regimen
1st	- Real-Time PCR SARS-CoV-2 of non-bronchoalveolar lavage (non-BAL)	SARS-CoV-2 (+)	corticosteroid therapy with dexamethasone (6 mg/day)+ remdesivir+ ceftriaxone
5th	Culture of non-BAL	*C. albicans*, *Aspergillus* spp.	Voriconazole+ ceftriaxone
6th	- GM antigen and PCR *Aspergillus* spp. of non-bronchoalveolar lavage (non-BAL) -GM antigen and PCR *Aspergillus* spp. of serum - Culture of non-BAL, MALDI-TOF and antifungal susceptibility testing	non-BAL: GM antigen (+), PCR *Aspergillus* spp. (+) GM antigen (-), PCR*Aspergillus* spp. (-) *Aspergillus niger*	Voriconazole+ ceftriaxone
11th	- FilmArray^®^, PneumoniaPanelplus(BIOFIRE, Biomerieux) of Non-bronchoalveolar lavage (non-BAL) - Culture, identification, and antibiotic susceptibility testing(Vitek2 Compact15, Biomerieux) of non-bronchoalveolar lavage (non-BAL)	- *Acinetobacter calcoaceticus-baumannii* complex (≥10^7^), *Streptococcus pneumoniae* (10^5^), KPC *Klebsiella pneumoniae* group (10^4^), *Staphylococcus aureus* (10^4^) Pandrug-resistant *A. baumannii*	Voriconazole+ meropenem+ colistin
18th	CT scan	three cavitary lesions with diameter up to 2.2 cm at upper and middle lobe of the right lung	Voriconazole+ liposomic amphotercinB+ meropenem+ colistin
38th	Culture of pleural effusion, identification and antibiotic susceptibility testing (Vitek2 Compact15, Biomerieux)	Pandrug-resistant *A. baumannii*	Meropenem+ colistin+ tigecycline

## References

[1] Thompson G.R., Young J.-A.H. (2021). Aspergillus infections. N. Engl. J. Med..

[2] Qin C., Zhou L., Hu Z., Zhang S., Yang S., Tao Y., Xie C., Ma K., Shang K., Wang W. (2020). Dysregulation of immune response in patients with Coronavirus 2019 (COVID-19) in Wuhan, China. Clin. Infect. Dis..

[3] Arastehfar A., Carvalho A., Van De Veerdonk F.L., Jenks J.D., Koehler P., Krause R., Cornely O.A., Perlin D.S., Lass-Flörl C., Hoenigl M. (2020). COVID-19 associated pulmonary aspergillosis (CAPA)—From immunology to treatment. J. Fungi.

[4] Somers E.C., Eschenauer G.A., Troost J.P., Golob J.L., Gandhi T.N., Wang L., Zhou N., Petty L.A., Baang J.H., Dillman N.O. (2020). Tocilizumab for treatment of mechanically ventilated patients with COVID-19. Clin. Infect. Dis..

[5] The RECOVERY Collaborative Group (2021). Dexamethasone in hospitalized patients with Covid-19. N. Engl. J. Med..

[6] Salmanton-García J., Sprute R., Stemler J., Bartoletti M., Dupont D., Valerio M., García-Vidal C., Falces-Romero I., Machado M., de la Villa S. (2021). COVID-19-associated pulmonary aspergillosis, March–August 2020. Emerg. Infect. Dis..

[7] Wahidi M.M., Lamb C., Murgu S., Musani A., Shojaee S., Sachdeva A., Maldonado F., Mahmood K., Kinsey M., Sethi S. (2020). American Association for Bronchology and Interventional Pulmonology (AABIP) Statement on the Use of Bronchoscopy and Respiratory Specimen Collection in Patients with Suspected or Confirmed COVID-19 Infection. J. Bronc. Interv. Pulmonol..

[8] Mohamed A., Rogers T.R., Talento A.F. (2020). COVID-19 associated invasive pulmonary aspergillosis: Diagnostic and therapeutic challenges. J. Fungi.

[9] Koehler P., Bassetti M., Chakrabarti A., Chen S.C.A., Colombo A.L., Hoenigl M., Klimko N., Lass-Flörl C., Oladele R.O., Vinh D.C. (2021). Defining and managing COVID-19-associated pulmonary aspergillosis: The 2020 ECMM/ISHAM consensus criteria for research and clinical guidance. Lancet Infect. Dis..

[10] Hamam J., Navellou J.-C., Bellanger A.-P., Bretagne S., Winiszewski H., Scherer E., Piton G., Millon L., Collaborative RESSIF group (2021). New clinical algorithm including fungal biomarkers to better diagnose probable invasive pulmonary aspergillosis in ICU. Ann. Intensiv. Care.

[11] Donnelly J.P., Chen S.C., Kauffman C.A., Steinbach W.J., Baddley J.W., Verweij P.E., Clancy C.J., Wingard J.R., Lockhart S.R., Groll A.H. (2020). Revision and Update of the Consensus Definitions of Invasive Fungal Disease from the European Organization for Research and Treatment of Cancer and the Mycoses Study Group Education and Research Consortium. Clin. Infect. Dis..

[12] Gangneux J.-P., Reizine F., Guegan H., Pinceaux K., Le Balch P., Prat E., Pelletier R., Belaz S., Le Souhaitier M., Le Tulzo Y. (2020). Is the COVID-19 Pandemic a Good Time to Include *Aspergillus* Molecular Detection to Categorize Aspergillosis in ICU Patients? A Monocentric Experience. J. Fungi.

[13] Apostolopoulou A., Garrigos Z.E., Vijayvargiya P., Lerner A.H., Farmakiotis D. (2020). Invasive Pulmonary Aspergillosis in Patients with SARS-CoV-2 Infection: A Systematic Review of the Literature. Diagnostics.

[14] Machado M., Valerio M., Álvarez-Uría A., Olmedo M., Veintimilla C., Padilla B., De La Villa S., Guinea J., Escribano P., Ruiz-Serrano M.J. (2021). Invasive pulmonary aspergillosis in the COVID-19 era: An expected new entity. Mycoses.

[15] Paramythiotou E., Dimopoulos G., Koliakos N., Siopi M., Vourli S., Pournaras S., Meletiadis J. (2021). Epidemiology and Incidence of COVID-19-Associated Pulmonary Aspergillosis (CAPA) in a Greek Tertiary Care Academic Reference Hospital. Infect. Dis. Ther..

[16] Van Arkel A.L.E., Rijpstra T.A., Belderbos H.N.A., Van Wijngaarden P., Verweij P.E., Bentvelsen R.G. (2020). COVID-19-associated Pulmonary Aspergillosis. Am. J. Respir. Crit. Care Med..

[17] Koehler P., Cornely O.A., Böttiger B.W., Dusse F., Eichenauer D.A., Fuchs F., Hallek M., Jung N., Klein F., Persigehl T. (2020). COVID-19 associated pulmonary aspergillosis. Mycoses.

[18] Lahmer T., Rasch S., Spinner C., Geisler F., Schmid R.M., Huber W. (2020). Invasive pulmonary aspergillosis in severe coronavirus disease 2019 pneumonia. Clin. Microbiol. Infect..

[19] Rutsaert L., Steinfort N., Van Hunsel T., Bomans P., Naesens R., Mertes H., Dits H., Van Regenmortel N. (2020). COVID-19-associated invasive pulmonary aspergillosis. Ann. Intensive Care.

[20] Bartoletti M., Pascale R., Cricca M., Rinaldi M., Maccaro A., Bussini L., Fornaro G., Tonetti T., Pizzilli G., Francalanci E. (2020). Epidemiology of Invasive Pulmonary Aspergillosis Among Intubated Patients With COVID-19: A Prospective Study. Clin. Infect. Dis..

[21] Verweij P.E., Gangneux J.P., Bassetti M., Brüggemann R.J.M., Cornely O.A., Koehler P., Lass-Flörl C., van de Veerdonk F.L., Chakrabarti A., Hoenigl M. (2020). Diagnosing COVID-19-associated pulmonary aspergillosis. Lancet Microbe.

[22] Balajee S.A., Marr K.A. (2006). Phenotypic and genotypic identification of human pathogenic aspergilli. Futur. Microbiol..

[23] Arendrup M., Friberg N., Mares M., Kahlmeter G., Meletiadis J., Guinea J., Andersen C., Arikan-Akdagli S., Barchiesi F., Chryssanthou E. (2020). How to interpret MICs of antifungal compounds according to the revised clinical breakpoints v. 10.0 European committee on antimicrobial susceptibility testing (EUCAST). Clin. Microbiol. Infect..

[24] Nasir N., Farooqi J., Mahmood S.F., Jabeen K. (2020). COVID-19-associated pulmonary aspergillosis (CAPA) in patients admitted with severe COVID-19 pneumonia: An observational study from Pakistan. Mycoses.

[25] Falces-Romero I., Ruiz-Bastián M., Díaz-Pollán B., Maseda E., García-Rodríguez J., Montero-Vega M.D., Romero-Gómez M.P., García-Bujalance S., Cendejas-Bueno E., Toro-Rueda C. (2020). Isolation of *Aspergillus* spp. in respiratory samples of patients with COVID-19 in a Spanish Tertiary Care Hospital. Mycoses.

[26] Alanio A., Dellière S., Fodil S., Bretagne S., Mégarbane B. (2020). Prevalence of putative invasive pulmonary aspergillosis in critically ill patients with COVID-19. Lancet Respir. Med..

[27] Armstrong R.A., Kane A.D., Kursumovic E., Oglesby F.C., Cook T.M. (2021). Mortality in patients admitted to intensive care with COVID-19: An updated systematic review and meta-analysis of observational studies. Anaesthesia.

[28] Fianchi L., Picardi M., Cudillo L., Corvatta L., Mele L., Trapè G., Girmenia C., Pagano L. (2004). *Aspergillus niger* Infection in Patients with Haematological Diseases: A Report of Eight Cases. Mycoses.

[29] Stanzani M., Vianelli N., Cavo M., Kontoyiannis D.P., Lewis R.E. (2019). Development and internal validation of a model for predicting 60-day risk of invasive mould disease in patients with haematological malignancies. J. Infect..

[30] Bassetti M., Vena A., Bouza E., Peghin M., Muñoz P., Righi E., Pea F., Lackner M., Lass-Flörl C. (2020). Antifungal susceptibility testing in Candida, Aspergillus and Cryptococcus infections: Are the MICs useful for clinicians?. Clin. Microbiol. Infect..

[31] Ergün M., Brüggemann R.J.M., Alanio A., Dellière S., van Arkel A., Bentvelsen R.G., Rijpstra T., Brugge S.V.D.S.-V.D., Lagrou K., Janssen N.A.F. (2021). Aspergillus test profiles and mortality in critically ill COVID-19 patients. J. Clin. Microbiol..

[32] Dimopoulos G., Almyroudi M.-P., Myrianthefs P., Rello J. (2021). COVID-19-associated pulmonary aspergillosis (CAPA). J. Intensive Med..

